# Downregulation of Annexin A1 by short hairpin RNA inhibits the osteogenic differentiation of rat bone marrow-derived mesenchymal stem cells

**DOI:** 10.3892/ijmm.2015.2243

**Published:** 2015-06-11

**Authors:** XINYUAN PAN, LIU PENG, GUOQIAN YIN

**Affiliations:** Department of Plastic and Aesthetic Surgery, The First Affiliated Hospital of Guangxi Medical University, Nanning, Guangxi 530021, P.R. China

**Keywords:** Annexin A1, bone marrow-derived mesenchymal stem cells, differentiation, osteogenic, extracellular signal-regulated kinase 1/2

## Abstract

Annexin A1 (ANX A1) is essential in cell differentiation and proliferation. However, the role of ANX A1 in bone marrow-derived mesenchymal stem cell (BM-MSC) osteogenic differentiation and proliferation remains unclear. To investigate whether endogenous ANX A1 influences BM-MSC proliferation and osteogenic differentiation, a stable ANX A1-knockdown cell line was generated using short hairpin RNA (shRNA). The proliferation rate of BM-MSCs was analyzed by 3-(4,5-Dimethylthiazol-2-yl)-2,5-diphenyltetrazolium bromide proliferation assay. Additionally, BM-MSCs were differentiated into osteoblasts and subsequently used to isolate total proteins to analyze the expression of ANX A1. Cell differentiation was assayed using Alizarin red S staining. The results revealed that the knockdown of ANX A1 in BM-MSCs exerts no apparent effect on the proliferation rate under normal conditions, however, following exposure to an osteogenic medium, downregulation of ANX A1 protected cells from the effect of osteogenic medium-induced inhibition of cell proliferation. Silencing ANX A1 with shRNA significantly inhibited the phosphorylation of extracellular signal-regulated kinase 1/2 and the expression of differentiation-associated genes (including runt-related transcription factor 2, osteopontin and osteocalcin) during osteogenesis and resulted in reduced differentiation of BM-MSCs. The results indicate the potential role of ANX A1 in the regulation of BM-MSC proliferation and osteogenic differentiation.

## Introduction

Bone marrow-derived mesenchymal stem cells (BM-MSCs) are multipotent adult cells that possess the potential to differentiate into a variety of cell types, including adipocytes, osteocytes, chondrocytes and neurons. The multipotential differentiation capacity makes this type of cell important candidates for tissue regenerative medicine (1). Over the past decade, various studies have indicated that the osteogenic differentiation of BM-MSCs is a complex process, regulated by a number of osteogenic transcription factors, such as peroxisome proliferator-activated receptor-γ, muscle segment homeobox 2 and runt-related transcription factor 2 (Runx2) (2,3). However, the molecular mechanism that controls the osteogenic differentiation of BM-MSCs is not well understood.

Annexin A1 (ANX A1), a member of the Annexin superfamily, plays an essential role in cell differentiation, proliferation and apoptosis. ANX A1 acts as a substrate for the epidermal growth factor (EGF) receptor tyrosine kinase and through its implication in the mitogen-activated protein kinase (MAPK)/extracellular signal-regulated kinase (ERK) glucocorticoid signaling pathway, performs important signaling functions in cell proliferation and differentiation (4). It has been reported that the phosphorylation of ANX A1 by EGF has an antiproliferative effect on macrophages due to the constitutive activation of the MAPK/ERK signaling pathway (5). The molecular mechanism behind the involvement of ANX A1 in cell differentiation is complex. ANX A1 is frequently dysregulated in various types of cancer and its expression is associated with tumor differentiation. The increased expression of ANX A1 has been reported in pancreatic cancer, colon adenocarcinoma and hepatocellular carcinoma (6–8). However, certain studies have demonstrated that the expression of ANX A1 is downregulated in thyroid, breast, and head and neck cancer (9–11). Furthermore, an increase of ANX A1 expression with differentiation has been reported to occur in normal epithelial cells (12). A recent study by Bizzarro *et al* (13) demonstrated that ANX A1 is upregulated during myoblast cell differentiation and a functional knockdown of the ANX A1 protein inhibits myogenic differentiation. Furthermore, an anomalous development of the craniofacial bone has been observed in ANX A1 null mice (14). This finding indicated that attenuation of ANX A1 may cause bone dysostosis. However, the functional role of ANX A1 in bone development remains unclear, and knowledge has been confined to a relatively small number of published findings.

ERK is the most extensively investigated MAPK family member, which is predominantly known for its role in the regulation of BM-MSC proliferation and osteogenic differentiation. The inhibition of extracellular matrix-induced ERK1/2 activation by PD98059 [an MAPK kinase (MEK) inhibitor] suppresses osteogenic differentiation of human MSCs (15). Additionally, ANX A1 specifically regulates the activity of the ERK cascade (16). Studies indicate that ANX A1 specifically regulates cell differentiation and proliferation through the ERK/MAPK signaling pathway (17). Thus, it may be hypothesized that aberrant activation of the ERK signaling pathway by the regulated expression of ANX A1 may influence BM-MSC osteogenic differentiation.

The role of ANX A1 in BM-MSC osteogenic differentiation and proliferation remains unclear. Therefore, in the present study, the inhibition of BM-MSC osteogenic differentiation as a result of ANX A1 downregulation was investigated.

## Materials and methods

### Reagents and antibodies

The following antibodies were used for western blot analysis: Rabbit anti-rat ANX A1 (ab65844) was obtained from Abcam (Cambridge, USA); and phospho-p44/42 MAPK (Thr202/Tyr204) rabbit mAb (catalogue no. 4370); p44/42 MAPK rabbit mAb (catalogue no. 4695); phospho-p38 MAPK (Thr180/Tyr182) rabbit mAb (catalogue no. 4511); p38 MAPK rabbit mAb (catalogue no. 8690); and glyceraldehyde 3-phosphate dehydrogenase (GAPDH) rabbit mAb (catalogue no. 5174) were obtained from Cell Signaling Technology, Inc. (Danvers, MA, USA). All the primary antibodies were used at a dilution of 1:1,000. The secondary anti-rabbit IRDye 800CW antibody was obtained from LI-COR Biosciences (Lincoln, NE, USA).

### Isolation and culture of BM-MSCs

The management of experimental animals used in the present study were according to the Regulations for the Administration of Affairs Concerning Experimental Animals (approved by the State Council of China and promulgated by decree no. 2 of the State Science and Technology Commission on November 14, 1988).

The BM-MSCs were isolated by adherence to plastic surfaces, as previously described (18). Briefly, thigh bones were isolated from 6 Sprague Dawley rats (body weight, 150–200 g; maintained under SPF raising conditions before surgery) and bone marrow containing mononuclear cells was flushed out with phosphate-buffered saline (PBS) using a syringe. The cell suspension was filtered through a 100-*μ*m strainer, and the cells were counted and centrifuged (500 x g for 10 min). The pellet was resuspended in Dulbecco’s modified Eagle’s medium (DMEM; Gibco Life Technologies, Carlsbad, CA, USA) supplemented with 10% fetal bovine serum (FBS; GE Healthcare Life Sciences, Logan, UT, USA), 100 U/ml penicillin and 100 *μ*g/ml streptomycin (Gibco Life Technologies). The harvested cells were seeded at a density of 1×10^6^/well in 6-well plates with the medium changed every 3 days. Cells were passaged upon reaching 70–80% confluence.

### ANX A1 knockdown via short hairpin RNA (shRNA)

Fourth-passage BM-MSCs were used for transfection. The shRNA (fluorescent dye-labeled shRNA; Shanghai GenePharma Co., Ltd., Shanghai, China) was designed to target the common sequence of rat ANX A1. The sense sequence of shRNA-ANXA1 was 5′-GCCT CACA ACCA TTGT GAAG T-3′, and a scrambled shRNA served as a negative control. The uniqueness of the designed shRNA was confirmed using the National Center for Biotechnology Information Basic Local Alignment Search Tool (http://blast.ncbi.nlm.nih.gov/Blast.cgi).

The BM-MSCs were plated at a density 5×10^3^/well in 96-well culture plates prior to transfection. After the cells had adhered for 24 h, the culture medium was changed to antibiotic-free serum media. Transducing lentiviral particles (Shanghai GenePharma Co., Ltd.) expressing ANX A1 shRNA or scrambled shRNA were added at the quantity that was calculated to reach an optimal multiplicity of infection of 80 units. After a 48-h incubation, the viral medium was removed and the cells were collected for further experiments. BM-MSCs expressing green fluorescent protein (GFP) reporter were analyzed under an Olympus CKX41SF fluorescence microscope (Olympus, Tokyo, Japan) 48 h post-transfection. The transfection efficiency was measured by flow cytometry (BD Accuri™ C5 flow cytometer; BD Biosciences, San Jose, CA, USA) 5 days post-transfection.

ANX A1 knockdown cells were designated as ANX A1-knockdown (ANX A1-KD) BM-MSCs and the cells that were infected with scrambled shRNA were termed negative control BM-MSCs.

### Osteogenic differentiation

To evaluate their osteogenic capacity, the cells (1×10^5^ cells/well) were seeded in 6-well plates and cultured in high-glucose DMEM supplemented with 10% FBS, 100 U/ml penicillin, 100 mg/ml streptomycin, 0.05 mM ascorbate, 1 *μ*M dexamethasone and 10 mM β-glycerophosphate for 18 days. Osteogenesis was confirmed by Alizarin red S (Solarbio, Beijing, China) staining and alkaline phosphatase (ALP) activity. The images were captured using an Olympus CKX41SF microscope (Olympus).

For Alizarin red S staining, the cells were seeded in 6-well plates and grown in osteogenic-inducing media until the indicated time point (10 and 18 days), when the cells were fixed in a solution of 4% formaldehyde. The cells were stained in 1 ml Alizarin red S solution for 20 min at room temperature and washed with ultra pure water. Alizarin red S-stained areas were evaluated via phase contrast microscopy (Olympus CKX41SF; Olympus). After staining, the cultures were incubated with 10% cetylpyridinium chloride at room temperature for 1 h to release the calcium-bound Alizarin. The absorbance of the released Alizarin red S was measured at a wavelength of 570 nm using a Multiskan MK3 spectrophotometer (Thermo Fisher Scientific, Inc., Rockford, IL, USA) (19).

BM-MSCs cultured in osteogenic-inducing media were collected from each well and assayed for ALP activity. The activity of ALP was measured by an Alkaline Phosphatase Activity Colorimetric assay kit (BioVision, Inc., Milpitas, CA, USA) according to the manufacturer’s instructions; the optical density (OD) was measured at 490 nm.

### 3-(4,5-Dimethylthiazol-2-yl)-2,5-diphenyltetrazolium bromide (MTT) proliferation assay

BM-MSC proliferation was measured using an MTT assay (Solarbio). Briefly, ~5×10^3^ cells were plated in 96-well plates for each appropriate time point (day 0, 1, 3, 5 and 7). The MTT solution was then added and maintained for 4 h at 37°C. Dimethyl sulfoxide (150 *μ*l) was added to each well for 10 min to solubilize the crystals. The OD value was measured at a wavelength of 490 nm using a spectrophotometer (Multiskan MK3; Thermo Fisher Scientific, Inc.).

### RNA isolation and reverse transcription-quantitative polymerase chain reaction (RT-qPCR)

Total cellular RNA extraction was performed using the RNAprep Pure Cell kit (Tiangen Biotech Co., Ltd., Beijing, China) according to the manufacturer’s instructions. To generate single stranded cDNA, RNA was reverse transcribed using a Revert Aid First Strand cDNA Synthesis kit (Thermo Fisher Scientific Inc.). PCR was performed with the ABI7500 system (Applied Biosystems Life Technologies, Foster City, CA, USA) using Fast Start Universal SYBR-Green Master (Roche Diagnostics, Indianapolis, IN, USA). The primer sequences used for RT-qPCR were as follows: Forward, 5′-ACCA GAAG AAGT ACGG AA-3′ and reverse, 5′-AACA ACGG CTAA GAGA TG-3′ for ANX A1; forward, 5′-GACA ACTT TGGC ATCG TGGA-3′ and reverse, 5′-ATGC AGGG ATGA TGTT CTGG-3′ for GAPDH; forward, 5′-CTGG GCTT AGAT GGAC-3′ and reverse, 5′-CTAT TATG GGCT GGGT-3′ for Runx2; forward, 5′-AACC AAGC GTGG AAAC-3′ and reverse, 5′-TGGA ACTC GCCT GACT-3′ for osteopontin (OPN); forward, 5′-GGCA GTAA GGTG GTGA A-3′ and reverse, 5′-CCTG GAAG CCAA TGTG-3′ for osteocalcin (OC).

The cycling conditions were as follows: Pre-incubation, 95°C for 5 min; PCR, 95°C for 30 sec and 58°C for 30 sec over 42 cycles; and final elongation, 72°C for 60 sec. Expression levels of the relative genes were calculated using the 2^−ΔΔCt^ method and GAPDH mRNA served as an internal control.

### Western blot analysis

Proteins were extracted from the BM-MSCs using radioimmunoprecipitation assay buffer (Beyotime Institute of Biotechnology, Beijing, China) and the total protein concentration was measured using a Bradford assay (Bio-Rad Laboratories, Inc., Hercules, CA, USA) according to the manufacturer’s instructions. Proteins (40–60 *μ*g/lane) were separated on a 10% sodium dodecyl sulfate-polyacrylamide gel electrophoresis and electrophoretically transferred to a polyvinylidene difluoride membrane (EMD Millipore, Billerica, MA, USA) for 1 h at 100 mA. Membranes were incubated in 3% bovine serum albumin/PBS-Tween for 1 h at room temperature. Primary antibodies were used at a dilution of 1:1,000 and applied by overnight (at least 12 h) incubation at 4°C. Subsequently, the membrane was incubated with a fluorescent-conjugated secondary antibody at a dilution of 1:10,000 for 1 h. Blots were analyzed using the Odyssey imaging system (LI-COR Biosciences).

### Statistical analysis

All data were expressed as the mean ± standard deviation. The statistical significance between groups was determined by Student’s t-test or one-way analysis of variance. Statistical analyses were performed using SPSS version 13.0 (SPSS, Inc., Chicago, IL, USA) and P<0.05 was considered to indicate a statistically significant difference.

## Results

### Successful knockdown of ANX A1 in rat BM-MSCs

**Figure 1:**
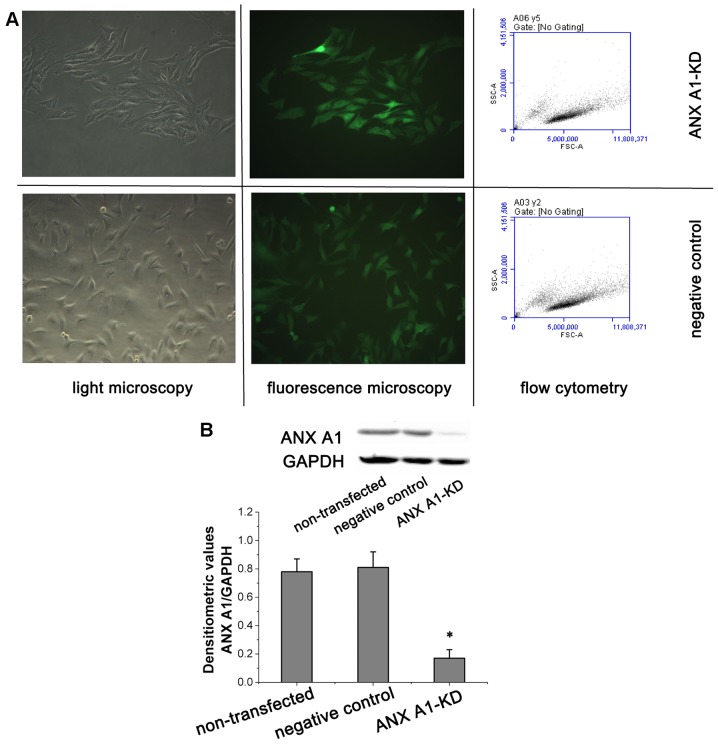
Effective shRNA-mediated suppression of the ANX A1 gene and protein. (A) BM-MSCs expressing green fluorescent protein reporter visualized by fluorescence microscopy compared with the total cells as determined by light microscopy (magnification, ×200). The transfection efficiency was detected by flow cytometry. (B) Western blot analysis of shRNA-mediated ANX A1-KD. Treatment with shRNA targeted to ANX A1 resulted in observable knockdown, whereas the negative control did not affect the ANX A1 protein levels in the BM-MSCs. The extent of ANX A1 knockdown was semi-quantitated using densitometry analysis (Quantity One statistical analysis). The ANX A1 semi-quantification was normalized to GAPDH expression. ^*^P<0.01 was determined using one-way analysis of variance, n=5 per group. ANX A1, Annexin A1; shRNA, short hairpin RNA; BM-MSCs, bone marrow-derived mesenchymal stem cells; KD, knockdown; GAPDH, glyceraldehyde 3-phosphate dehydrogenase. ^*^P<0.01 vs. the negative control cells.

To investigate the effect of ANX A1 on proliferation and differentiation of BM-MSCs, a stable knockdown cell line was generated using lentiviral particles. The microphotograph in Fig. 1A depicts GFP-expressing cells 2 days after infection. Flow cytometric analysis confirmed that BM-MSCs achieved transfection efficiencies of ≤80% for the ANX A1-KD group and ≤75% for the negative control group (Fig. 1A).

Downregulation of ANX A1 at the transcription and translation levels was confirmed by SYBR-Green RT-qPCR and western blot analysis. On average, treatment with shRNA targeted to ANX A1 resulted in a 68.1% reduction in ANX A1 mRNA levels (P<0.01; n=5) and a 76.2% decrease in ANX A1 protein levels (P<0.01, n=5) in ANX A1-KD BM-MSCs when compared with scrambled shRNA (Fig. 1B). No significant difference was observed in the reduction of protein levels between the utransfected BM-MSCs and the negative control BM-MSCs (P<0.05).

### Effect of ANX A1 on BM-MSC proliferation

**Figure 2:**
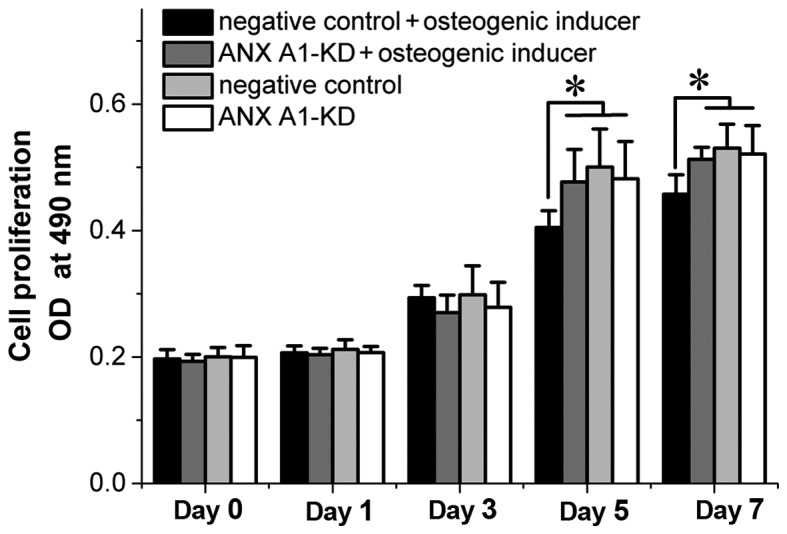
Effect of downregulating ANX A1 on bone marrow-derived mesenchymal stem cell proliferation during osteogenic differentiation. OD was measured at 490 nm for the 3-(4,5-Dimethylthiazol-2 -yl)-2,5-diphenyltetrazolium bromide proliferation assay. ^*^P<0.05, n=5 per group. ANX A1, Annexin A1; OD, optical density; KD, knockdown.

Expression levels of ANX A1 have been found to affect cell proliferation *in vitro* (20,21). To determine whether the knockdown of ANX A1 affected BM-MSC proliferation, the proliferation rate of BM-MSCs was analyzed by MTT assay. Although ANX A1-KD cells cultured in normal conditions exhibited a marginal decrease in the proliferation rate, no significant difference was identified between the ANX A1-KD and negative control cells. However, when cells were cultured in the osteogenic-inducing media, the negative control cells exhibited an inhibited proliferation rate at day 5 and 7 when compared with the ANX A1-KD cells (P<0.05; Fig. 2).

### ANX A1 expression is upregulated during the later stages of osteogenic differentiation

**Figure 3:**
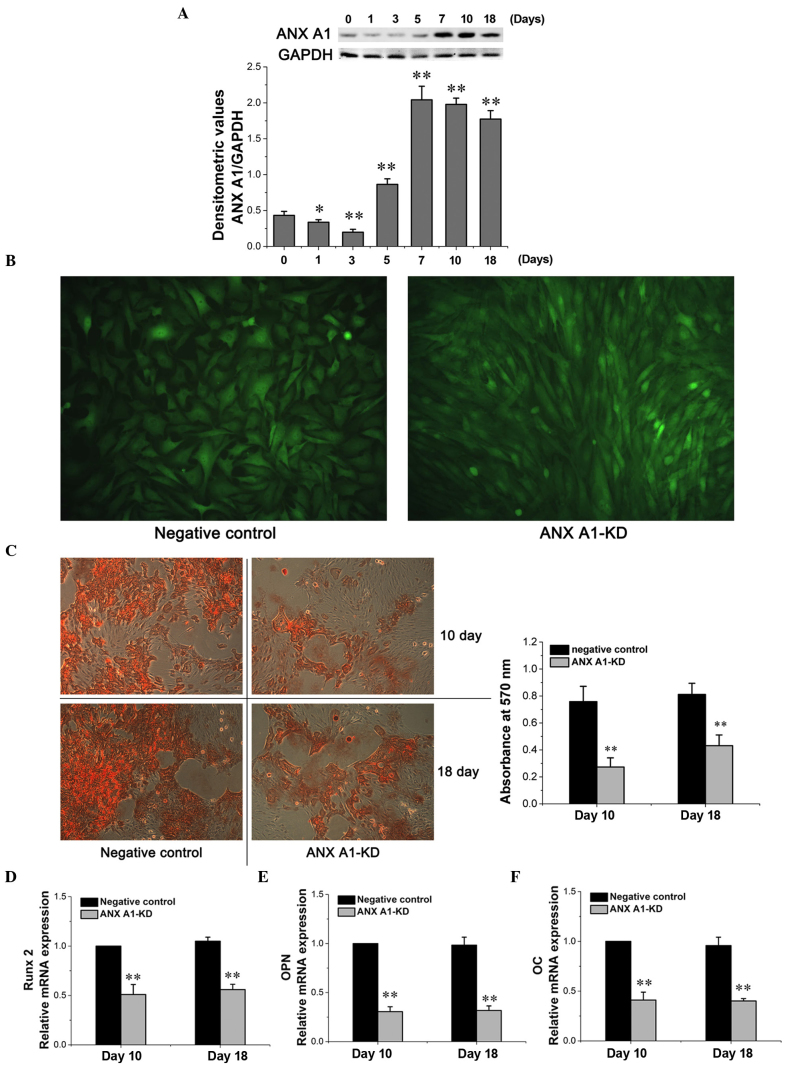
Knockdown of ANX A1 by shRNA resulted in reduced differentiation of BM-MSCs. (A) Analysis of ANX A1 protein expression during osteogenesis. GAPDH served as an internal control and the quantitative analysis of ANXA1 expression was performed by densitometry. The amount of ANXA1 was normalized to the amount of GADPH. Quantitative analysis of the ANX A1 protein expression is presented in the bar chart. ^*^P<0.05 and ^**^P<0.01 vs. day 0 (n=5 per group). (B) The shape of the negative control BM-MSCs became angular during osteogenesis compared with the ANX A1-KD BM-MSCs (magnification, ×200). (C) Analysis of Alizarin red S staining and quantification of matrix mineralization (magnification, ×100). Knockdown of ANX A1 significantly inhibited calcified matrix synthesis. (D–F) Silencing of ANX A1 by shRNA significantly suppressed Runx2, OPN and OC gene expression. ^**^P<0.01 vs. the negative control, as determined by one-way analysis of variance, n=5 per group. KD, knockdown; ANX A1, Annexin A1; shRNA, short hairpin RNA; BM-MSC, bone marrow-derived mesenchymal stem cells; GAPDH, glyceraldehyde 3-phosphate dehydrogenase; Runx2, runt-related transcription factor 2; OPN, osteopontin; OC, osteocalcin.

The expression of ANX A1 in the negative control BM-MSCs during differentiation was analyzed to determine how endogenous ANX A1 is regulated throughout BM-MSC osteogenic differentiation. For osteogenic differentiation, the cells were cultured with osteogenic-inducing media. BM-MSC differentiation into osteoblasts was induced and these cells were used to analyze the expression of ANX A1 during differentiation. Following osteogenic-inducing media treatment, a marginal decrease in the expression of ANX A1 was apparent at day 1 and 3, and subsequently an ongoing increase was observed from day 5 to 18 (P<0.01; Fig. 3A). Therefore, it was hypothesized that the expression of ANX A1 may be involved in the later stages of osteogenic differentiation.

### Downregulation of ANX A1 inhibits BM-MSC osteogenic differentiation

To analyze whether ANX A1 downregulation leads to inhibition of BM-MSC differentiation into osteoblasts, BM-MSCs were infected with the shRNA-ANX A1 plasmid. In addition, BM-MSCs were infected with scrambled shRNA that served as a negative control. The ANX A1-KD BM-MSCs and the negative control BM-MSCs were induced to differentiate into mature osteoblasts, and the Alizarin red S staining was performed to investigate the maturity of the osteoblasts.

Following 10 days of exposure to osteogenic-inducing media, the negative control cells became angular in shape, with increased cell extensions observed during osteogenesis, whereas the ANX A1-KD BM-MSCs were spindle-shaped (Fig. 3B). The ALP activity of ANX A1-KD BM-MSCs cultured in osteogenic medium was significantly lower than that in the negative control BM-MSCs (day 10, 0.224±0.074 vs. 0.646±0.126 nmol/min/mg and day 18, 0.319±0.039 vs. 0.691±0.096 nmol/min/mg; P<0.01, n=5).

Alizarin red S staining was performed after 10 and 18 days of culturing in the osteogenic-inducing media. The negative control BM-MSCs showed matrix mineralization with more intense Alizarin red S staining when compared with the ANX A1-KD BM-MSCs (Fig. 3C). Quantitative analysis of mineralization by measuring the absorbance of Alizarin red S at 570 nm revealed a significant difference between the two groups on days 10 and 18 (P<0.01; Fig. 3C), indicating that the knockdown of ANX A1 inhibited BM-MSC osteogenic differentiation.

To further investigate the influence of ANX A1 knockdown on the BM-MSCs osteogenic differentiation, the expression of Runx2 mRNA and its downstream target genes, OPN and OC was evaluated. RT-qPCR analysis revealed that silencing ANX A1 using shRNA significantly suppressed the Runx2, OPN and OC gene expression after 10 and 18 days of culturing in osteogenic-inducing media (P<0.01; Fig. 3D–F).

### Knockdown of ANX A1 decreases ERK1/2 phosphorylation during osteogenic differentiation

**Figure 4:**
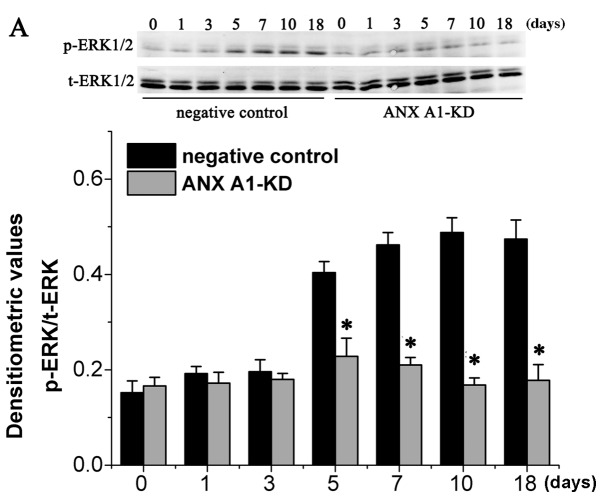

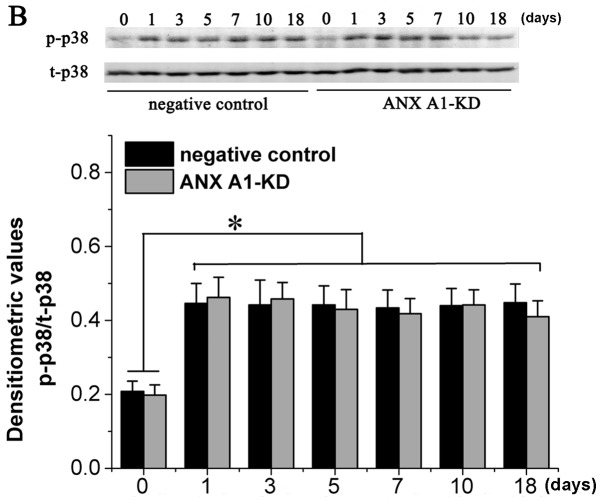
Effect of silencing ANX A1 on the phosphorylation of ERK1/2 and p38 MAPK during osteogenic differentiation. (A) The expression level of p-ERK and t-ERK1/2 proteins was analyzed by western blotting. The phosphorylation of ERK1/2 in the negative control cells was higher from day 5 to 18 compared with that of the ANX A1-KD BM-MSCs (^*^P<0.01 vs. the negative control cells, n=5 per group). (B) The phosphorylation rate of p38 MAPK was increased over the entire 18-day period during osteogenic differentiation compared with day 0 (^*^P<0.01, n=5 per group), however no difference was identified between ANX A1-KD BM-MSCs and negative control BM-MSCs. Densitometry was performed using Quantity One statistical analysis and P<0.01 was determined using one-way analysis of variance. p, phosphorylated; ERK1/2, extracellular signal-regulated kinase 1/2; ANX A1, Annexin A1; BM-MSC, bone marrow-derived mesenchymal stem cell; MAPK, mitogen-activated protein kinase.

It has been demonstrated that the ERK-MAPK signaling pathway is pivotal during MSC differentiation (22). Therefore, the phosphorylation status of ERK1/2 in BM-MSCs, which had been stimulated with osteogenic-inducing media from day 1 to 18, was investigated. Phosphorylation of ERK1/2 was initially elevated in the negative control BM-MSCs following exposure to osteogenic-inducing media for 5 days, and remained highly phosphorylated at day 7, 10 and 18 (Fig. 4A). However, the phosphorylation of ERK1/2 in the ANX A1-KD BM-MSCs was significantly inhibited when compared with the negative control BM-MSCs (P<0.01).

In an attempt to elucidate whether the knockdown of ANX A1-mediated inhibition of BM-MSC osteogenic differentiation involves the activation of p38 MAPK, the status of p38 phosphorylation during the course of differentiation of BM-MSCs into osteoblasts was analyzed. The results presented in Fig. 4B demonstrate that the phosphorylation of p38 MAPK was significantly elevated and remained highly phosphorylated from day 1 to 18; however, the absence of ANX A1 did not alter the p38 MAPK phosphorylation.

Taken together, these results support the hypothesis that the silencing of ANX A1 with shRNA significantly reduces the differentiation of BM-MSCs by inhibiting the activation of ERK1/2, but not that of p38 MAPK.

### Involvement of ERK1/2 activation in osteogenic differentiation

**Figure 5:**
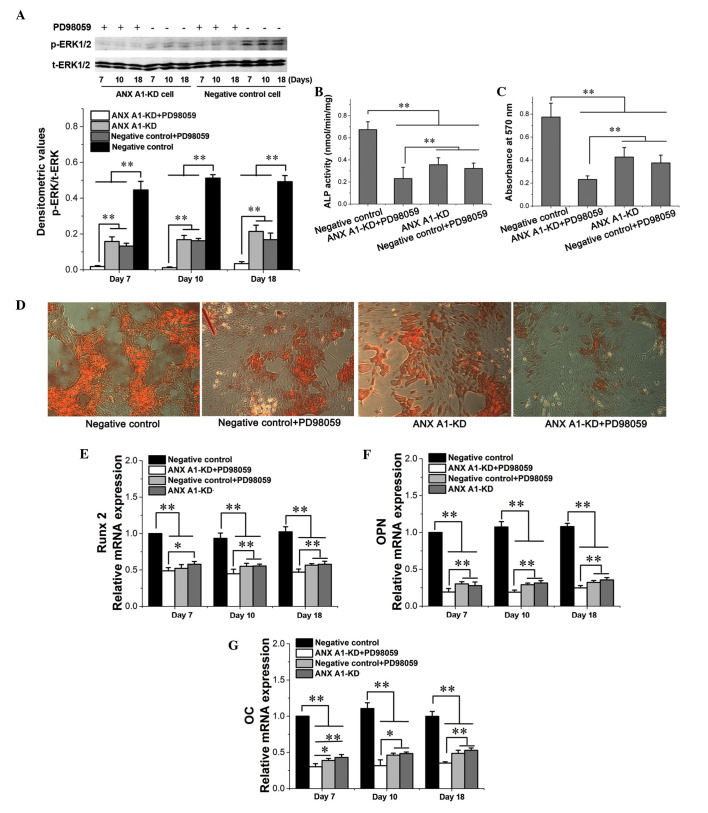
Effect of PD98059 on the ability of BM-MSCs to undergo osteogenic differentiation. (A) Treatment of BM-MSCs with PD98059 produced a significant inhibition of ERK1/2 phosphorylation. (B and C) Results of ALP activity and the spectroscopic calcium assay at day 18. PD98059 treatment resulted in a significant decrease of calcified matrix synthesis and ALP activity (^**^P<0.01). (D) Osteogenic differentiation demonstrated by staining with Alizarin red S 18 days after induction reveals that PD98059 treatment inhibits mineralization (magnification, ×100). (E–G) Quantitative reverse transcription polymerase chain reaction analysis of Runx2, OPN and OC gene expression. Treatment of cells with PD98059 produced a significant inhibition of the expression levels of those genes (^**^P<0.01). Statistical significance was determined by one-way analysis of variance. ^*^P<0.05 and ^**^P<0.01, n=5 per group. p, phosphorylated; KD, knockdown; ANX A1, Annexin A1; BM-MSC, bone marrow-derived mesenchymal stem cell; ERK1/2, extracellular signal-regulated kinase 1/2; ALP, alkaline phosphatase; Runx2, runt-related transcription factor 2; OPN, osteopontin; OC, osteocalcin.

Due to previous reports describing that ANX A1 modulates the ERK signaling pathway at a proximal site (16) and knockdown of ANX A1 inhibits ERK1/2 activation (23), an inhibitor of MEK/ERK, PD98059, was used in the current study to mimic the inhibitory effect caused by knockdown of ANX A1 on ERK1/2 activation. In the present study, negative control cells or ANX A1-KD cells were treated with osteogenic-inducing media supplemented with or without 20 *μ*M PD98059. Treatment of negative control cells with PD98059 resulted in a significant inhibition of ERK1/2 activity (P<0.01). In addition, a further decrease of ERK1/2 activity was observed in ANX A1-KD cells treated with PD98059 (P<0.01; Fig. 5A). A significant decrease of calcified matrix synthesis and ALP activity was observed in the cell culture with PD98059, as well as in the ANX A1-KD cell culture with osteogenic-inducing media alone (P<0.01; Fig. 5B–D). Furthermore, it was observed that ANX A1-KD cells in combination with PD98059 treatment further inhibited the ALP activity and calcified matrix synthesis when compared with the ANX A1-KD cells without PD98059 treatment (P<0.01; Fig. 5B and C).

The effect of PD98059 on the osteogenic gene expression was also assessed. Consistent with the data obtained from ERK1/2 activity, ANX A1-KD cells together with PD98059 treatment resulted in a reduced decrease of Runx2, OPN and OC mRNA expression when compared with ANX A1-KD cells alone (P<0.01; Fig. 5E–G). In addition, treatment of the negative control cells with 20 *μ*M PD98059 produced a significant suppression of Runx2, OPN and OC mRNA expression when compared with the untreated negative control cells (P<0.01), however, no significant difference was noted when compared with the group of ANX A1-KD cells without PD98059 treatment (P<0.05; Fig. 5E–G).

Taken together, these data demonstrate that the activation of ERK1/2 is necessary for osteogenic differentiation and that treatment with PD98059 may enhance the inhibitory effect of ANX A1 knockdown on the osteogenic differentiation of BM-MSCs.

## Discussion

ANX A1, the first characterized member of the Annexin super-family, is found in numerous cells and tissues, including the lungs, bone marrow and intestine (24). Annexins are associated with the cell membrane or cytoskeleton in a calcium-dependent manner. Accumulating evidence has demonstrated that the expression level of ANX A1 is involved in cell proliferation and differentiation (25). The present study was undertaken to investigate the effects of ANX A1 on BM-MSC proliferation and osteogenic differentiation. The results demonstrated that the knockdown of ANX A1 with shRNA in BM-MSCs resulted in reduced osteogenic differentiation. It was also found that the knockdown of ANX A1 caused a reduction in ERK1/2 phosphorylation and osteogenic gene expression during osteogenic differentiation. Furthermore, downregulation of ANX A1 was observed to decrease the rate of proliferation reduction following BM-MSC incubation in osteogenic-inducing media.

Although the expression of ANX A1 has been associated with various types of cellular differentiation (25), to the best of our knowledge, little previous evidence exists implicating ANX A1 in the differentiation of BM-MSCs into osteoblasts. In the present study, it was found that the expression of ANX A1 marginally decreased on day 1 and 3 following exposure to an osteogenic medium, however, it was increased markedly on day 5 and remained elevated up to day 18. To confirm the role of ANX A1 in osteogenic differentiation, BM-MSCs were transfected with shRNA-ANXA1. The results demonstrate that knockdown of ANX A1 significantly inhibited the expression of the osteogenic genes (Runx2, OPN and OC) and resulted in reduced differentiation of BM-MSCs into osteocytes, implying that the upregulation of ANX A1 in the later stages of differentiation may be required for further osteogenic differentiation.

The differentiation of BM-MSCs into osteoblasts is a complex process involving the interplay of numerous effectors that regulate, positively and negatively, a network of signaling pathways. The ERK, p38 and c-Jun N-terminal kinase (JNK) MAPKs are cell signaling pathways that are pivotal in cell differentiation. The role of p38 MAPK in osteogenic differentiation remains unclear, and previous studies gave controversial results. Certain studies claim that activation of ERK1/2, but not JNKs or the p38 MAPKs, promotes osteogenesis (26), whereas others state that p38 MAPKs promote osteogenic differentiation (27). However, studies have confirmed that the ERK signaling pathway affects osteogenesis via different mechanisms during the differentiation process (28). ANX A1 specifically modulates the ERK signaling cascade at an upstream site. Increasing the expression of ANX A1 leads to constitutive activation of ERK1/2 kinase (16). A recent study found that ERK activation was significantly decreased in ANX A1 KD-DU145 cells (23). However, it has been reported that p38 MAPK function, upstream of dexamethasone-induced ANX A1 synthesis, and the p38 MAPK inhibitor, SB203580 prevent dexamethasone-induced ANX A1 expression (29). In the present study, a sustained phosphorylation of ERK1/2 was observed from day 5 to 18 during negative control BM-MSC differentiation into osteoblasts. This phosphorylation was correlated with the expression of ANX A1, which was upregulated in the later stages of differentiation. Furthermore, knockdown of ANX A1 significantly inhibited the phosphorylation of ERK1/2 during BM-MSC osteogenic differentiation. Although activation of the p38 MAPK signaling cascade has been associated with BM-MSC osteogenic differentiation (27), the present study demonstrates that knockdown of ANX A1 exerts no apparent effect on altering p38 MAPK phosphorylation. Therefore, it was hypothesized in the current study that ANX A1 regulation of ERK1/2 phosphorylation may be involved in osteogenic differentiation.

In order to further confirm the involvement of the ERK1/2 signaling pathway in BM-MSC osteogenic differentiation, the effect of PD98059 on the ability of negative control BM-MSCs to undergo osteogenic differentiation was analyzed in the present study. It was found that blocking ERK1/2 phosphorylation resulted in decreased mineralization, ALP activity and expression of osteogenic genes (Runx2, OPN and OC) when BM-MSCs were incubated in osteogenic-inducing media. This result is consistent with earlier observations made by Jaiswal *et al* (30). Although knockdown of ANX A1 resulted in a decrease of ERK1/2 phosphorylation, the inhibition was not complete. Therefore, ANX A1-KD cells were treated with PD98059, to establish whether it would potentiate the inhibitory effects of ANX A1 knockdown on osteogenic differentiation. As expected, a more marked reduction in mineralization and osteogenic gene expression was observed in the ANX A1-KD cells treated with PD98059, when compared with the group of ANX A1-KD cells not treated with PD98059. These results further support the hypothesis that osteogenic differentiation of BM-MSCs requires activation of ERK1/2 and that the inhibitory effect of ANX A1 knockdown on BM-MSC osteogenic differentiation involves ERK1/2 inhibition.

ANX A1 regulates ERK signaling pathway activation and inhibits cyclin D1 expression, therefore reducing cell proliferation (20). Evidence reveals that ANX A1 functions as an inhibitor of signal transduction pathways that leads to cell proliferation in JACRO cells (21) and lymphocytes (31). However, the effects of over-expression of ANX A1 in rapidly proliferating hepatocytes (7), as well as human foreskin fibroblasts (32) indicates that the underlying mechanism is more complex. Although downregulation of ANX A1 has been traditionally associated with increased cellular proliferation, the data from the present study indicates that the proliferation rate of ANX A1-KD BM-MSCs was not significantly increased under normal conditions. However, when negative control cells were cultured in the osteogenic-inducing media, a decrease in cell proliferation was observed, when compared with that of the ANX A1-KD cells. A previous study proposed that the proliferation of ANX A1^+/+^ cells were inhibited by dexamethasone in a concentration-dependent manner (0.01–1 *μ*M), whereas the ANX A1^−/−^ cells were not significantly affected at these dexamethasone concentrations (21). In addition, one study demonstrated that the addition of osteogenic-inducing media to human BM-MSCs inhibited cell proliferation (33). The MTT data obtained in the current study indicates that the negative control BM-MSCs over-expressing the ANX A1 protein have a reduced proliferation rate from day 5 to 7, this reduction is likely to be associated with the over-expression of ANX A1 and the osteogenic medium, which contains 1 *μ*M dexamethasone. Therefore, it is proposed that, under the exposure of osteogenic-inducing media, downregulation of ANX A1 may protect cells from the effect of osteogenic medium-induced inhibition of cell proliferation.

In conclusion, the present study revealed that the knockdown of ANX A1 in BM-MSCs significantly effects the proliferation rate under osteogenic-inducing media treatment. Additionally, silencing ANX A1 using shRNA significantly inhibits the phosphorylation of ERK1/2 during osteogenesis and results in reduced osteogenic differentiation of BM-MSCs. These data identify that ANX A1-mediated ERK1/2 activation is involved in BM-MSC proliferation and osteogenic differentiation. Furthermore, these results may have important implications with regard to bone development and remodeling.
